# Predictors of Mortality Amongst Recipients of Implantable Cardioverter Defibrillators for Secondary Prevention of Cardiac Arrest

**Published:** 2007-10-22

**Authors:** Sunil K Agarwal, Ish Singla, Haitham Hreybe, Samir Saba

**Affiliations:** Cardiac Electrophysiology Section of the University of Pittsburgh Medical Center, Pittsburgh, PA

**Keywords:** Implantable Cardioverter Defibrillators, Cardiac Arrest, Mortality, Predictors

## Abstract

**Background:**

Implantable Cardioverter-defibrillators (ICD) reduce mortality in survivors of cardiac arrest (CA).  We investigated the predictors of mortality after ICD implantation in survivors of CA.

**Methods:**

Retrospective review of clinical records and social security death index of all patients who received an ICD in a preexisting database of survivors of CA at the University of Pittsburgh Medical Center was performed. Multivariate analyses using the Cox proportional hazard model were performed with backward elimination to identify independent predictors of the time to death, and Kaplan-Meier curves were plotted.

**Results:**

Eighty patients (64 men) with a mean age of 64.4±12.5 years were followed for 4.7±2.3 years after ICD implantation. Survival rates were 93.8%, 65% and 50% at 1, 5, and 10 years, respectively. Independent predictors of time to death were determined to include age (hazard ratio (HR) = 1.91 per 10-year increase, p = 0.003), serum creatinine ≥ 1.3 mg/dL (HR = 2.56, p = 0.004), and QRS width >120 ms (HR = 5.14, p = 0.012).

**Conclusions:**

In this sample of ICD recipients secondary to CA, older age, elevated serum creatinine, and wider QRS duration were independent predictors of mortality. The presence of more than one risk factor in the same patient was associated with higher mortality rates. Whether interventions such as biventricular pacing can offset this increase risk of death warrants further investigation.

Sudden cardiac death accounts for 50% of all cardiac related mortality in the developed countries and half of these deaths occur outside hospitals [1]. Fatal arrhythmias are the leading causes of cardiac arrests (CA), mostly due to either delayed or unavailable defibrillation therapy. Implantable cardioverter-defibrillators (ICDs) provide timely electrical shocks in the face of near-fatal arrhythmias. Hence, they prolong survival of individuals, who already had a CA, as shown in secondary prevention ICD trials [2-4].  Despite the salutary effects of ICDs, defibrillator recipients have higher total mortality compared to the general population, which may be related to their underlying cardiac condition or other comorbidities [5,6].

Multiple studies have looked at the predictors of all-cause mortality as well as cardiovascular or arrhythmic deaths in ICD recipients [7,8], with inconsistent results. Many of these studies focused on patients with predominantly primary indication for ICD implantation. Factors such as detectable troponin T levels conferred a poor prognosis despite the ICD [9]. In other patient populations, the ICD did not improve survival in patients with higher blood pressure [9] or in those who were on therapy with statins [8].

We evaluated the predictors of total mortality in CA survivors, after implantation of an ICD, with the primary goal of determining and possibly averting non-arrhythmic causes of death that may still claim patient lives after the implantation of the defibrillator.

## Methods

### Study Subjects and Settings

We analyzed a preexisting database of all patients admitted to the University of Pittsburgh Medical Center with a concomitant diagnosis of acute myocardial infarction (AMI) and CA (n = 48). The Institutional Review Board of the University of Pittsburgh approved this study. In that database, those patients were matched by age, gender, and left ventricular ejection fraction (LVEF) to patients with CA outside the context of AMI (n = 48). Of the overall cohort (n=96), only patients implanted with an ICD (n=80) were included in this analysis.

Patients were followed up for a mean period of 4.7±2.3 years. Mortality status was ascertained from electronic medical record or social security mortality index.  Data on co-morbidities, clinical laboratory values, electrocardiogram, echocardiograms, and family history were obtained by examining the electronic medical records.

### Statistical Methods

Means (± standard deviations) and percentages were use to describe continuous and categorical variables, respectively. First, univariate analyses were done using the log rank test for equality across strata for categorical variables and the univariate Cox proportional hazard regression model for continuous variables. Multivariate Cox proportional hazard regression linear models were used including variables with a p value ≤ 0.1, using backward elimination. Other predictors of mortality shown in published literature were similarly tested using backward elimination. Variables selected for multivariate analyses were used to build a final model after checking for interactions and proportionality assumptions. Kaplan-Meier curves were plotted for variables found significant on multivariate Cox model by dichotomizing markers according to clinical relevance. Finally, the effect of the presence of one or more mortality predictor was evaluated by plotting Kaplan-Meier survival curves and using the log rank test. P values ≤ 0.05 were considered statistically significant. All statistical analyses were performed on SAS statistical package (version 9.1.3).

## Results

**Figure 1:**
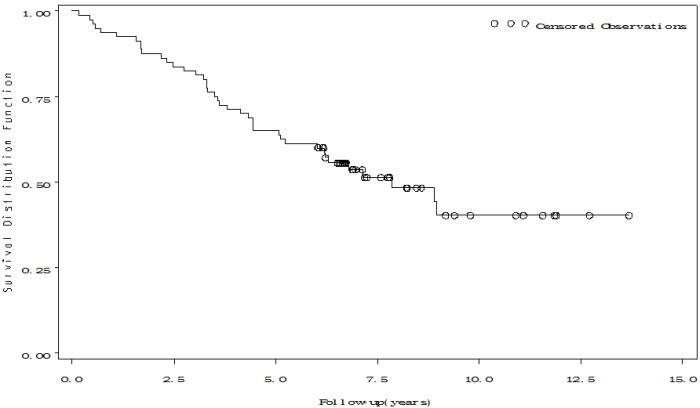
Kaplan-Meier Survival Curves the whole Cohort of ICD Recipients Secondary to Sudden Cardiac Arrest (n=80)

Eighty patients (64 men) with a mean age of 64.4±12.5 years underwent ICD implantation. Of those, 47 patients had only CA, while the remaining had both AMI and CA. Fifty percent of the cohort (n = 40) died during follow-up, with a mean survival of 5.9±3.1 years (Range 0.16-13.7 years). Kaplan-Meier survival curve for the overall cohort is shown in Figure 1. The 1-year, 5-year and 10-year survival rates after CA and ICD implantation were 93.8% , 65%, and 50%, respectively.

Baseline characteristics differed significantly between patients who died during follow-up and those who survived, as shown in Table 1. In brief, older age, higher serum creatinine, wider QRS complex, presence of coronary artery disease, a higher white blood cell count, lower platelet and creatinine phosphokinase counts, and a higher occurrence of atrial fibrillation were more prevalent in those patients who died.

Backward selection methods yielded only age, serum creatinine, and QRS width as independent predictors of the time from ICD implantation to death during follow-up. The multivariate Cox proportional hazard model including these 3 variables is shown in Table 2. There was no statistical interaction between any of these independent predictors of time to death in our population. Also, the test of proportionality was not significant neither collectively nor for each of the final variables in the model.

**Figure 2:**
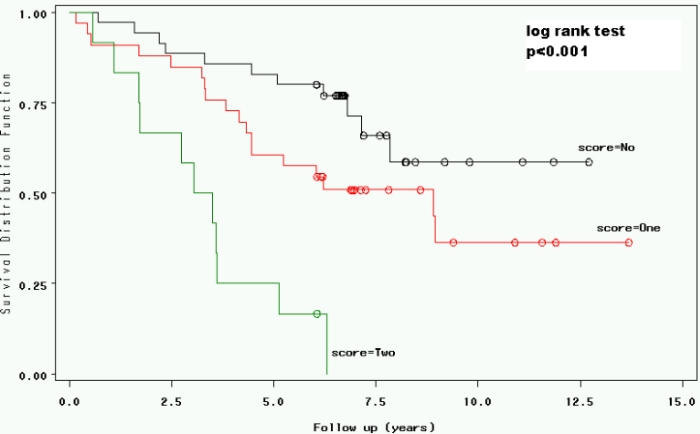
Kaplan Meier Curves Showing the Influence of the Presence of zero (n=35), one (n=33), and two or more (n=12) risk factors (Age > 70 years, Serum Creatinine >1.3 mg/dL, QRS Duration > 120ms) on Survival.

A higher risk profile was defined by dichotomizing serum creatinine at  >1.3mg/dL, the QRS width at > 120 ms, and age at > 70 years. As shown in Figure 2, there was a dose-dependent increase in the risk of mortality with the increase in number of these risk factors in a given patients (p<0.001).

## Discussion

This study shows that higher age, elevated serum creatinine levels, and a wider QRS complex at the time of ICD implantation are independent predictors of time to death in survivors of CA.  Our study cohort is closely comparable to the population of patients studied in the AVID trial. The unadjusted 3-year survival rates in AVID was 64.1% in the control arm and 75.4% in the ICD arm. Our 3-year survival rate is 82.5%, which is consistent with the fact that the ICD confers a survival benefit in this high-risk population of CA survivors. Patients with 2 or more risk factors in our cohort had however a 3-year survival rate of 50%, which is even lower than that of the no-ICD arm of AVID. This finding underscores the nature of competing causes of death in any individual patient whereby, despite a significant reduction in the risk of arrhythmic death with the ICD, patients are still likely to die sooner of other causes if they are older, have renal insufficiency, have a wider QRS complex which may exacerbate heart failure, or any combination of these risk factors. Whether the presence of 2 or more of these risk factors in one patient would overwhelm any benefit from the ICD deserves to be investigated. Until such data is available, survivors of CA remain indicated for ICD implantation regardless of age and QRS width.

Identifying baseline predictors of overall mortality can help reduce total mortality in ICD recipients if reversible causes of mortality are identified. Others have reported that women are less likely than men to benefit from ICD implantation [10]. Our results, however, do not confirm this finding and this is in keeping with larger published studies [7,11,12]. Also, similar to other studies, higher age [11,12] and worse kidney function [11,13,16] were independent predictors of mortality in our cohort.

In keeping with the findings of published ICD trials [17-19], longer QRS duration was associated with decreased survival in our cohort.  Prolonged QRS is associated with higher levels of ventricular dyssynchrony, with reduction in the rate of rise of ventricular pressure, and with reduction in diastolic filling times [20,21]. Biventricular pacing in a select population of patients with wide QRS complex, low LVEF, and advanced symptoms of heart failure has been shown to decrease mortality and morbidity. Whether a survival benefit is conferred by biventricular pacing in survivors of CA with wide QRS complex remains to be proven.

### Limitations

Our current study has a number of limitations. First, it is a retrospective study, with a small sample size, mostly consisting of white males with secondary indication for ICD implantation. Extrapolating our results to other patient populations of different ethnicities or implanted with ICDs for primary prevention of CA may not be feasible. Second, although all-cause mortality is an important cardiovascular end-point, disease specific mortality data would have provided valuable information.

In conclusion, this hospital-based cohort of CA survivors has higher survival compared to a historical control from the non-ICD arm of the AVID trial, except when other risk factors including advanced age, wide QRS complex, and the presence of renal insufficiency are documented. Future studies investigating the effect of aggressive management of renal dysfunction and the potential role of biventricular pacing in correcting ventricular conduction abnormalities on total mortality in this patient population may be warranted.

## Figures and Tables

**Table 1:**
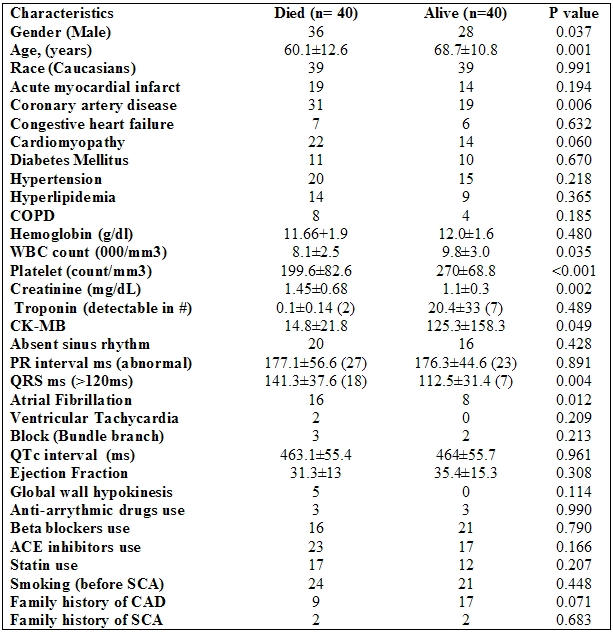
Characteristics of Study Subjects Prior to ICD Implantation (n=80) by Mortality Status

COPD=Chronic obstructive pulmonary disease, SCA=Sudden cardiac arrest, CAD= Coronary artery disease. P value refers to log rank p for categorical variables and Cox proportional model p for continuous variable

**Table 2:**
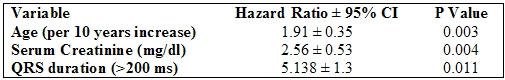
Multivariate Cox Proportional Hazard Ratio for Final* Variables

*These variables were selected using backward elimination from a set of variables with p<0.1 in univariate analysis and shown to be important mortality predictor in current literature.
